# Significance of secretory leukocyte peptidase inhibitor in pleural fluid for the diagnosis of benign asbestos pleural effusion

**DOI:** 10.1038/s41598-021-92289-7

**Published:** 2021-06-21

**Authors:** Takumi Kishimoto, Yoko Kojima, Nobukazu Fujimoto

**Affiliations:** Department of Medicine, Okayama Rosai Hospital, 1-10-25 Chikko Midorimachi, Minami-ku, Okayama, 702-8055 Japan

**Keywords:** Biomarkers, Oncology

## Abstract

Secretory leukocyte peptidase inhibitor (SLPI) is a biomarker present in the respiratory tract that protects against tissue destruction and aids in wound healing. We examined whether SLPI in pleural effusion can be used to distinguish benign asbestos pleural effusion (BAPE) from early-stage malignant pleural mesothelioma (MPM) and other diseases. We measured the levels of SLPI, hyaluronic acid (HA), soluble mesothelin-related peptides (SMRP), CCL2, galectin-3, and CYFRA21-1 in 51 patients with BAPE, 37 patients with early-stage MPM, 77 patients with pleural effusions due to non-small-cell lung cancer (LCa), and 74 patients with other pleural effusions. SLPI levels in the pleural fluid of patients with BAPE were significantly lower than those in patients with MPM, LCa, and other pleural effusions (p < 0.0001). The area under the curve (AUC) for SLPI’s ability to distinguish BAPE from MPM was 0.902, with a sensitivity of 82.4% and a specificity of 86.5%. This AUC was not only favourable but was better than the AUC for the ability of CYFRA21-1 to distinguish BAPE (0.853). The combination of SLPI and CYFRA21-1 achieved an AUC of 0.965 for the differentiation between BAPE and MPM. Pleural fluid SLPI as well as CYFRA21-1 and HA is useful as a biomarker to diagnose BAPE, which needs to be distinguished from early-stage MPM.

## Introduction

Benign asbestos pleural effusion (BAPE), an inflammatory lesion of the pleura caused by asbestos fibres, was first reported in 1964 by Eisenstadt^1^. The underlying mechanism of BAPE has yet to be elucidated. However, it is a condition that must be differentiated from early-stage malignant pleural mesothelioma (MPM). BAPE is an exudative pleural effusion, and its diagnosis is based on a history of occupational exposure to asbestos, findings of pleural plaque on imaging, and the elimination of other possible causes using pleural fluid markers, cytology, and pleural biopsy.

Early-stage lesions of MPM often present with pleural effusion only; however, as the disease progresses, imaging findings such as pleural rind patterns may suggest a malignant tumour. In early-stage lesions, neoplastic pleural thickening is typically not present, making it difficult to differentiate from other diseases, particularly BAPE. Because the diagnosis of early-stage lesions and surgical treatment can improve prognoses, when patients with a history of asbestos exposure present with pleural effusion, BAPE should be considered for a differential diagnosis from MPM.

In patients who present with pleural effusion, such as macroscopic and neoplastic pleural thickening, with no abnormal findings on computed tomography (CT) of the chest, accurate identification of the lesion site can be challenging, even with thoracoscopic biopsy. When sufficient tumour tissue cannot be obtained from a biopsy site, it can be difficult to make a histopathological diagnosis. In such instances, the detection of p16 gene deletion using fluorescence in situ hybridization (FISH) may be useful^2,3^. In addition, the detection of BRCA1-associated protein 1 deletion in cell nuclei as well as deletion of the p16 gene is reportedly important^4^.

In the diagnosis of MPM, elevated levels of hyaluronic acid (HA)^5,6^ and soluble mesothelin-related peptides (SMRP)^7,8^ in pleural fluid are reportedly useful for its differentiation from other diseases. Secretary leukocyte peptidase inhibitor (SLPI) is a serine protease inhibitor found in the respiratory tract and in the mucous of the cervical canal, nasal discharge, and saliva. Its physiological function is associated with wound healing and the prevention of tissue destruction, and it is considered a promising biomarker of acute renal impairment following heart surgery^9^.

In BAPE, which is an inflammatory lesion of the visceral pleura caused by asbestos fibres, the significance of SLPI in pleural fluid has yet to be confirmed. Here, we measured SLPI levels as a biomarker for BAPE, which is difficult to differentiate from early-stage MPM, and obtained results superior to those involving other markers.

## Methods

We analysed the pleural fluid of 51 patients with a definitive diagnosis of BAPE, 37 patients with early-stage MPM (diagnosed by histology from pleural biopsy; 31 with epithelial MPM and six with sarcoma-type conditions), 77 patients with malignant pleural effusions due to non-small-cell lung cancer (LCa) (diagnosed by cytological and histological examinations), 27 patients with heart failure (HF), and 47 patients with bacterial pleurisy (IF) diagnosed at Okayama Rosai Hospital between 2015 and 2019. The 74 patients with HF and IF were considered “other” patients.

BAPE can be diagnosed, according to the criteria of Epler et al.^10^ in individuals with a history of occupational exposure to asbestos and in whom the presence of pleural effusion can be confirmed. However, a diagnosis of BAPE is based on the presence of an exudate with no cause other than asbestos exposure and deaminase and carcinoembryonic antigen in pleural fluid, as well as cytology, pleural plaque findings and, depending on the patient, pleural biopsy results^11^.

In this study, we analysed biomarkers of MPM, such as HA, SMRP, CCL2, galectin-3 and CYFRA21-1, in pleural effusion and compared these markers with SLPI. We measured SLPI using a human SLPI Quantikine enzyme-linked immunosorbent assay (ELISA), HA using latex coagulating nephelometry, SMRP using Lumipulse CLEIA, CCL2 using a human MCP-1 ELISA Kit (PromoKine), galectin-3 using a human galectin-3 ELISA kit (PromoKine), and CYFRA21-1 using a colorimetric fixed quantity assay.

Significant differences between each disease were determined using a nonparametric Kruskal–Wallis test in accordance with Dunn’s post hoc test, and a p value < 0.05 was deemed a significant difference. The reliability of each marker was evaluated using a receiver operating characteristic (ROC) curve. The cut-off value was determined based on the curve, and the specificity and sensitivity were calculated. Analyses were performed using R and GraphPad Prism statistical software.

All participants provided written informed consent before inclusion in the study. This study was approved by the 3rd Reserch Ethics Committe of Okayama Rosai Hospital on February 26, 2015 (No.113-1). All study procedures were carried out in accordance with the principles of the Declaration of Helsinki.

### Consent for publication

All participants approve this publication.

## Results

### Biomarkers for BAPE, MPM, LCa and other diseases

**Figure 1:**
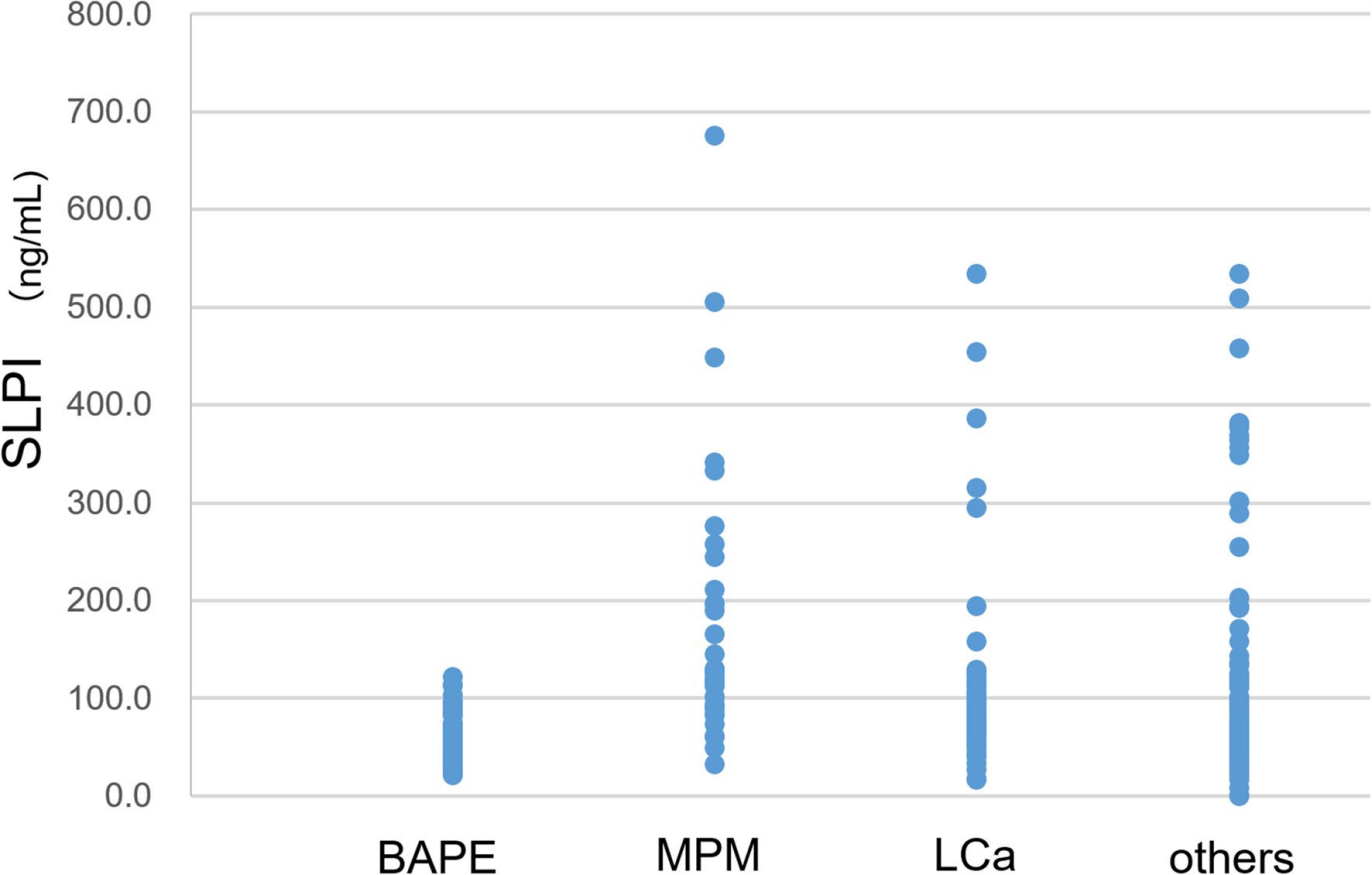
SLPI levels in pleural effusion from patients with BAPE, MPM, LCA, HF and IF. *SLPI* secretory leukocyte peptidase inhibitor, *BAPE* benign asbestos pleural effusion, *MPM* malignant pleural mesothelioma, *LCA* lung cancer, *HF* heart failure, *IF* bacterial pleurisy.

As shown in Fig. 1, the SLPI level was 57.8 ± 25.7 ng/mL in patients with BAPE, which was significantly lower than that in patients with MPM (173.0 ± 134.5 ng/mL) (p < 0.0001), LCa (103.5 ± 85.7 ng/mL) (p < 0.01), and other diseases (124.8 ± 12.9 ng/mL) (p < 0.05).

**Figure 2:**
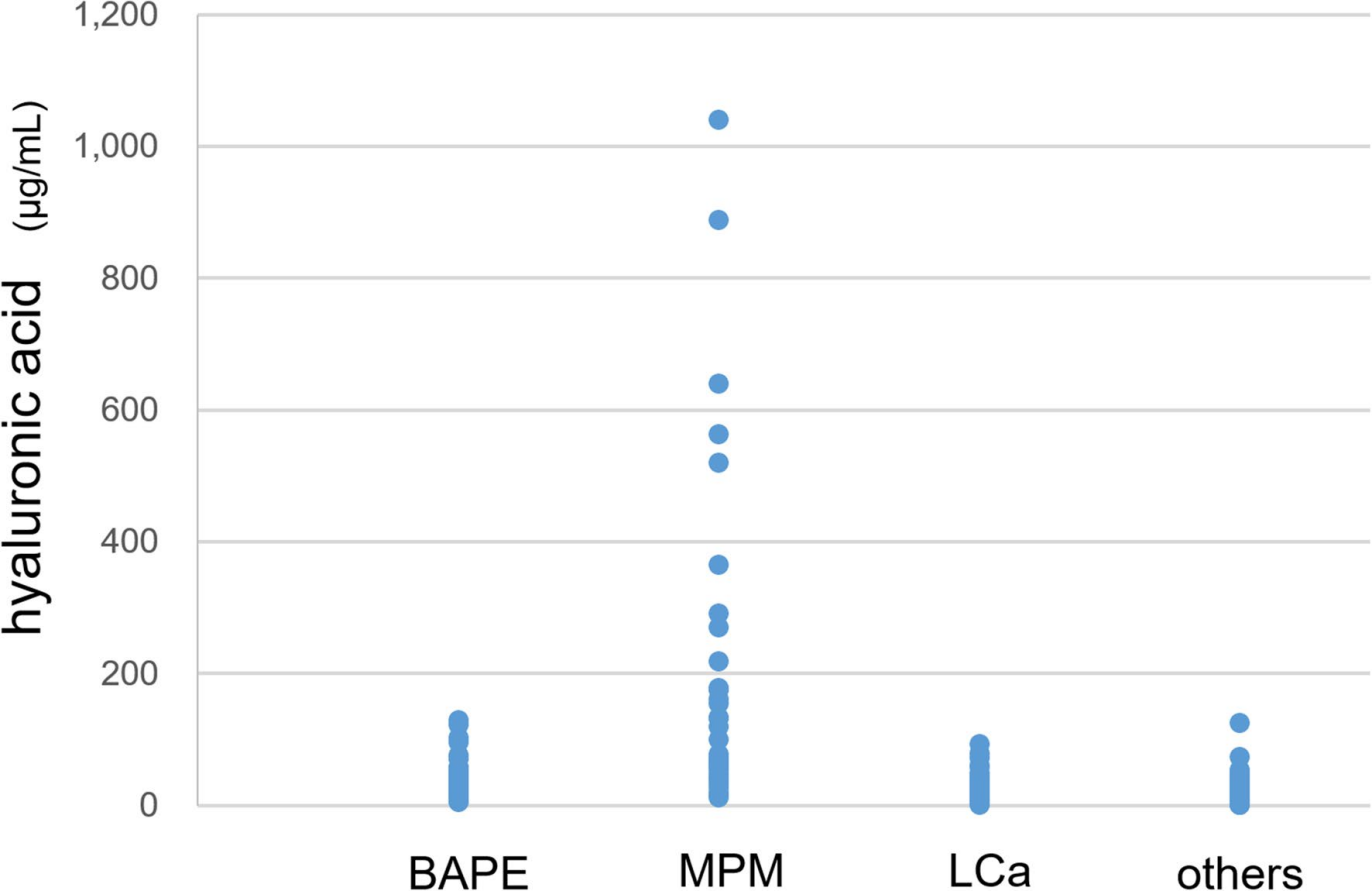
HA levels in pleural effusion from patients with BAPE, MPM, LCA and other diseases. *HA* hyaluronic acid, *BAPE* benign asbestos pleural effusion, *MPM* malignant pleural mesothelioma, *LCA* lung cancer.

The HA level was 38.7 ± 4.0 μg/mL in patients with BAPE, which was significantly lower than that in patients with MPM (192.4 ± 40.4 μg/mL) (p < 0.01). On the other hand, the HA level was 25.1 ± 2.1 μg/mL in patients with LCa and 21.5 ± 2.0 μg/mL in patients with other diseases, indicating a significantly (p < 0.05) higher level in patients with BAPE (Fig. 2).

**Figure 3:**
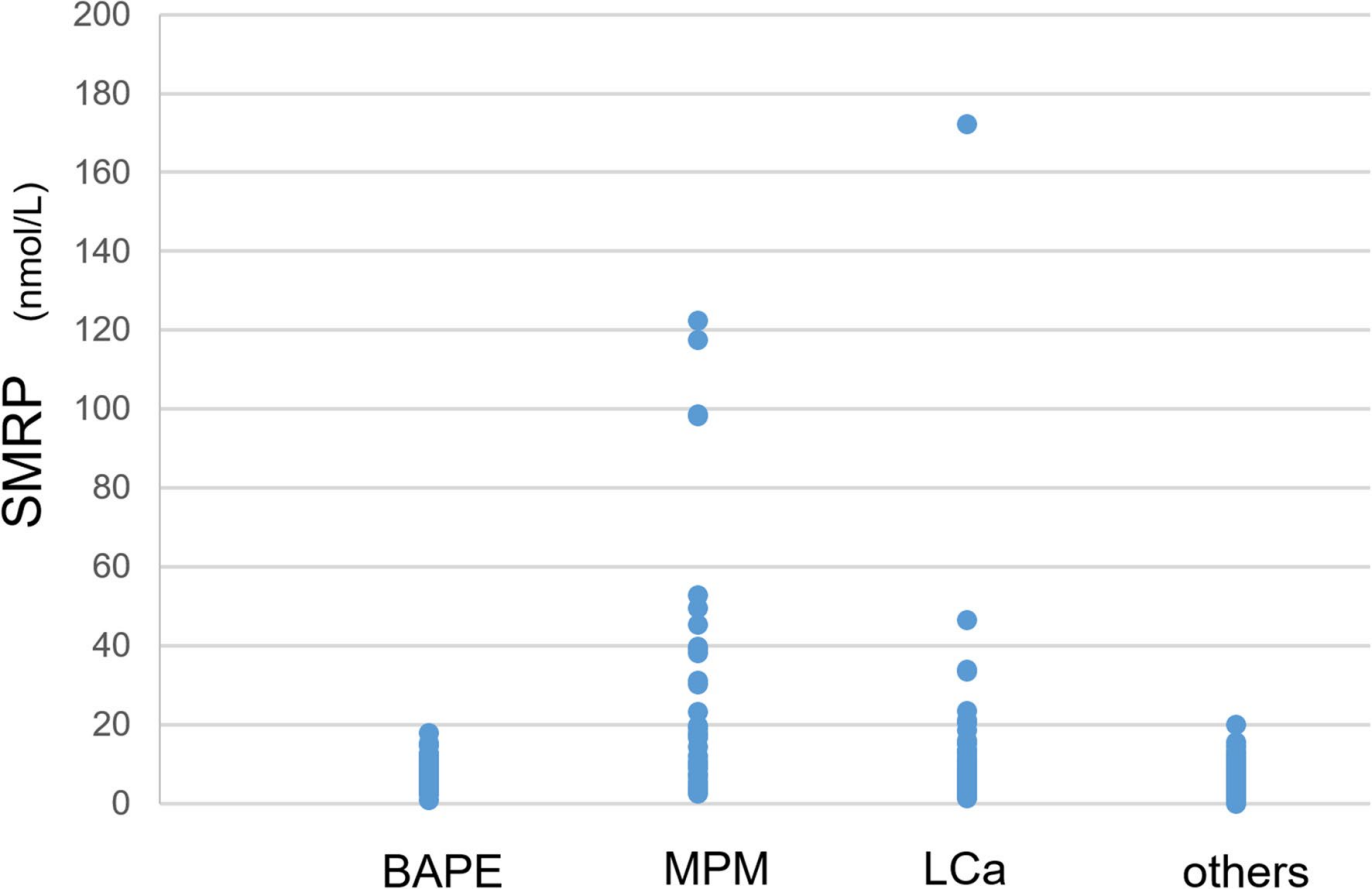
SMRP levels in pleural effusion from patients with BAPE, MPM, LCA, and IF. *SMRP* soluble mesothelin-related peptides, *BAPE* benign asbestos pleural effusion, *MPM* malignant pleural mesothelioma, *LCA* lung cancer, *IF* bacterial pleurisy.

The SMRP level was 7.5 ± 0.5 nmol/L in patients with BAPE, which was significantly lower than that in patients with MPM (28.5 ± 5.5 nmol/L) (p < 0.01) and significantly higher than that in patients with other diseases (5.6 ± 0.4 nmol/L) (p < 0.05). On the other hand, in LCa patients, the SMRP level was roughly comparable at 11.0 ± 2.5 nmol/L, with no significant difference observed (p < 0.512) (Fig. 3).

**Figure 4:**
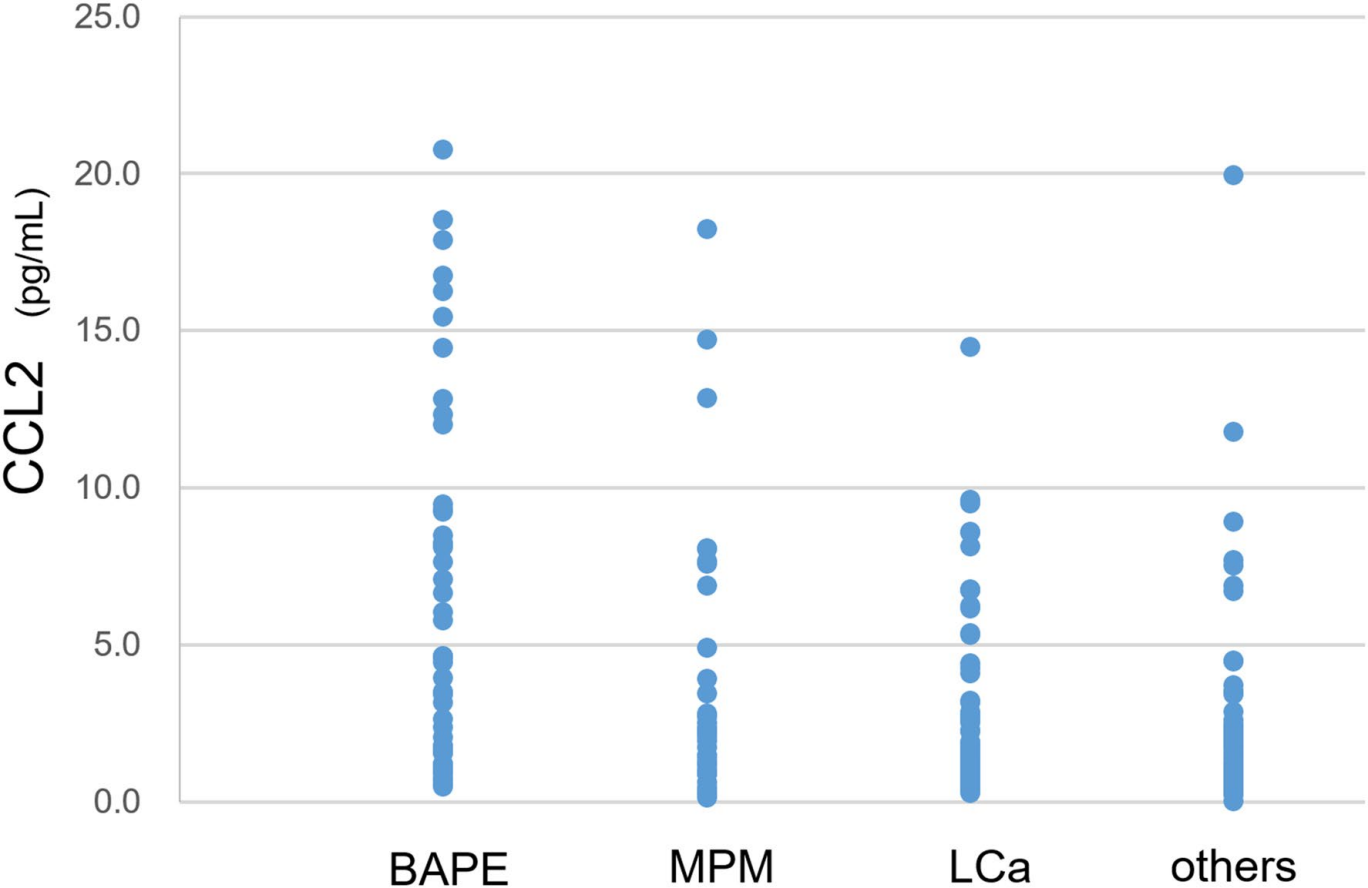
CCL2 levels in pleural effusion from patients with BAPE, MPM, LCA and other diseases. *CCL2* C–C motif chemokine 2, *BAPE* benign asbestos pleural effusion, *MPM* malignant pleural mesothelioma, *LCA* lung cancer.

The CCL2 level was 6.1 ± 0.8 pg/mL in patients with BAPE, which was significantly higher than that in patients with MPM (3.7 ± 0.7 pg/mL), LCa (2.5 ± 0.3 pg/mL), and other diseases (2.0 ± 0.3 pg/mL) (all p < 0.05) (Fig. 4). However, the data differed according to the histological type, with a level of 1.5 pg/mL for patients with epithelioid MPM and 9.5 pg/mL for patients with sarcomatoid MPM. This can difference be attributed to the fact that three of six patients with sarcomatoid MPM had relatively high levels (between 12.9 and 18.2 pg/mL).

**Figure 5:**
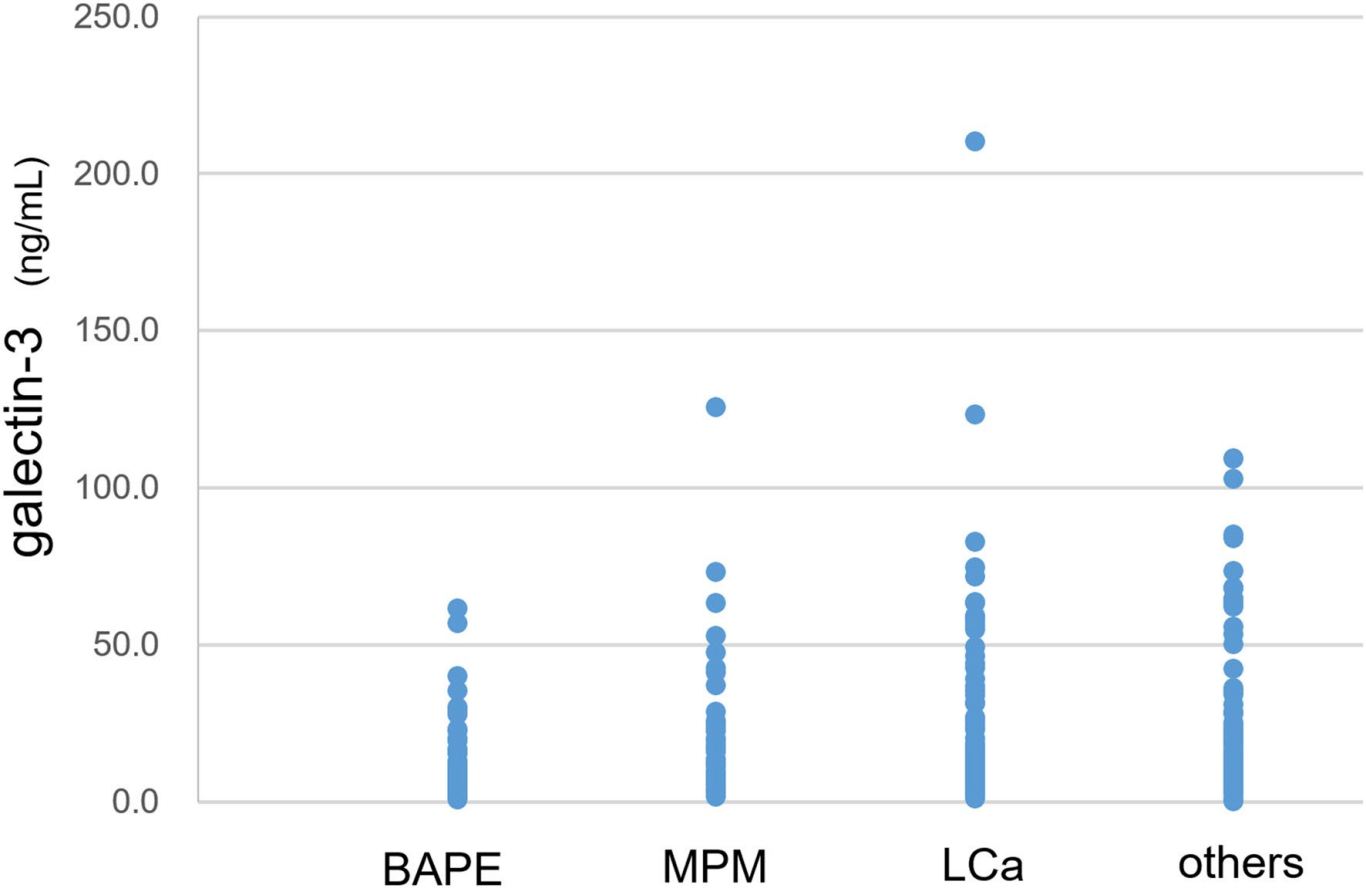
Galectin-3 levels in pleural effusion from patients with BAPE, MPM, LCA and other diseases. *BAPE* benign asbestos pleural effusion, *MPM* malignant pleural mesothelioma, *LCA* lung cancer.

Regarding other differential markers, the measured level of galectin-3 was 6.1 ± 0.8 ng/mL in patients with BAPE, which was significantly lower than that in patients with MPM (23.7 ± 4.0 ng/mL), LCa (26.1 ± 3.6 ng/mL), and other diseases (23.4 ± 2.6 ng/mL) (all p < 0.05) (Fig. 5).

The CYFRA21-1 level was 20.3 ± 2.3 ng/mL in patients with BAPE, 162.0 ± 35.8 ng/mL in patients with MPM, 164 ± 31.9 ng/mL in patients with LCa, and 21.1 ± 3.9 ng/mL in patients with other diseases. That is, while these levels were significantly lower in patients with BAPE than in patients with MPM and LCa (p < 0.03), they were significantly higher than in patients with other diseases (p < 0.0001).

### Biomarkers to differentiate BAPE from MPM

**Figure 6:**
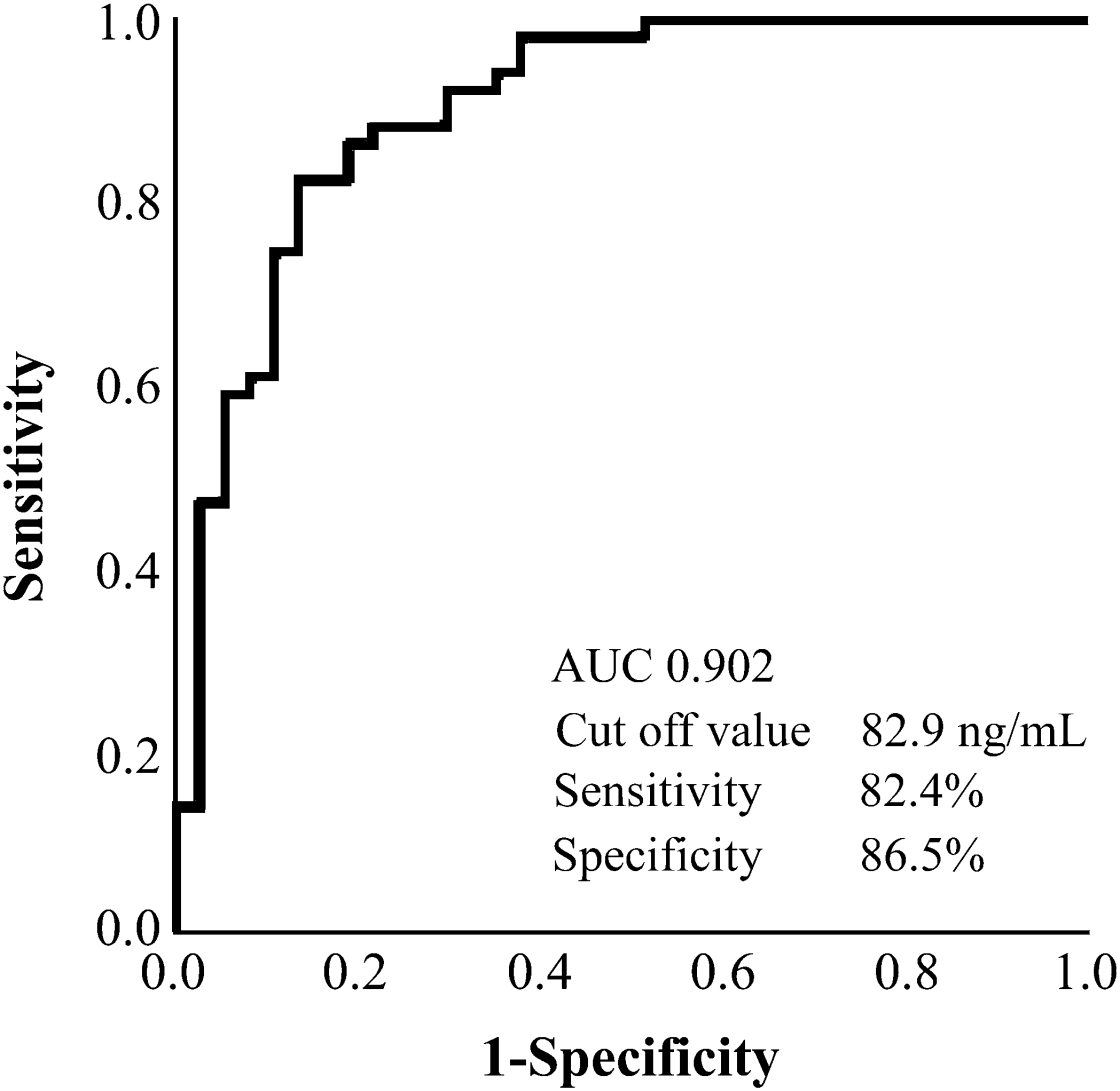
Receiver operating characteristic curve of SLPI for the differential diagnosis between BAPE and MPM. *BAPE* benign asbestos pleural effusion, *MPM* malignant pleural mesothelioma, *SLPI* secretory leukocyte peptidase inhibitor (SPSS Statistics V26 for IBM Japan (https://www.ibm.com/jp-ja/products/spss-statistics)).

**Figure 7:**
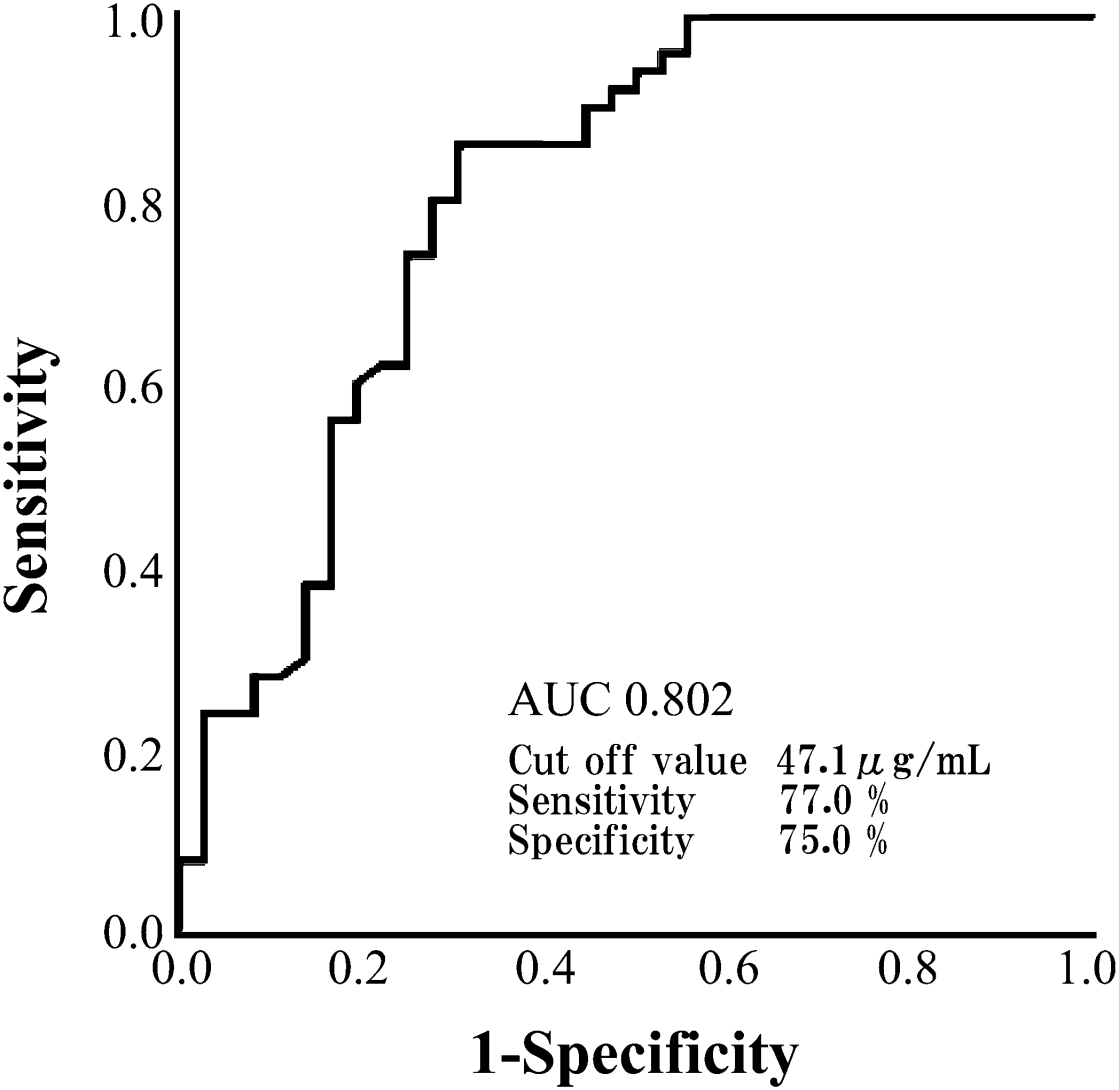
Receiver operating characteristic curve for HA for the differential diagnosis between BAPE and MPM. *BAPE* benign asbestos pleural effusion, *MPM* malignant pleural mesothelioma, *HA* hyaluronic acid (SPSS Statistics V26 for IBM Japan (https://www.ibm.com/jp-ja/products/spss-statistics)).

**Figure 8:**
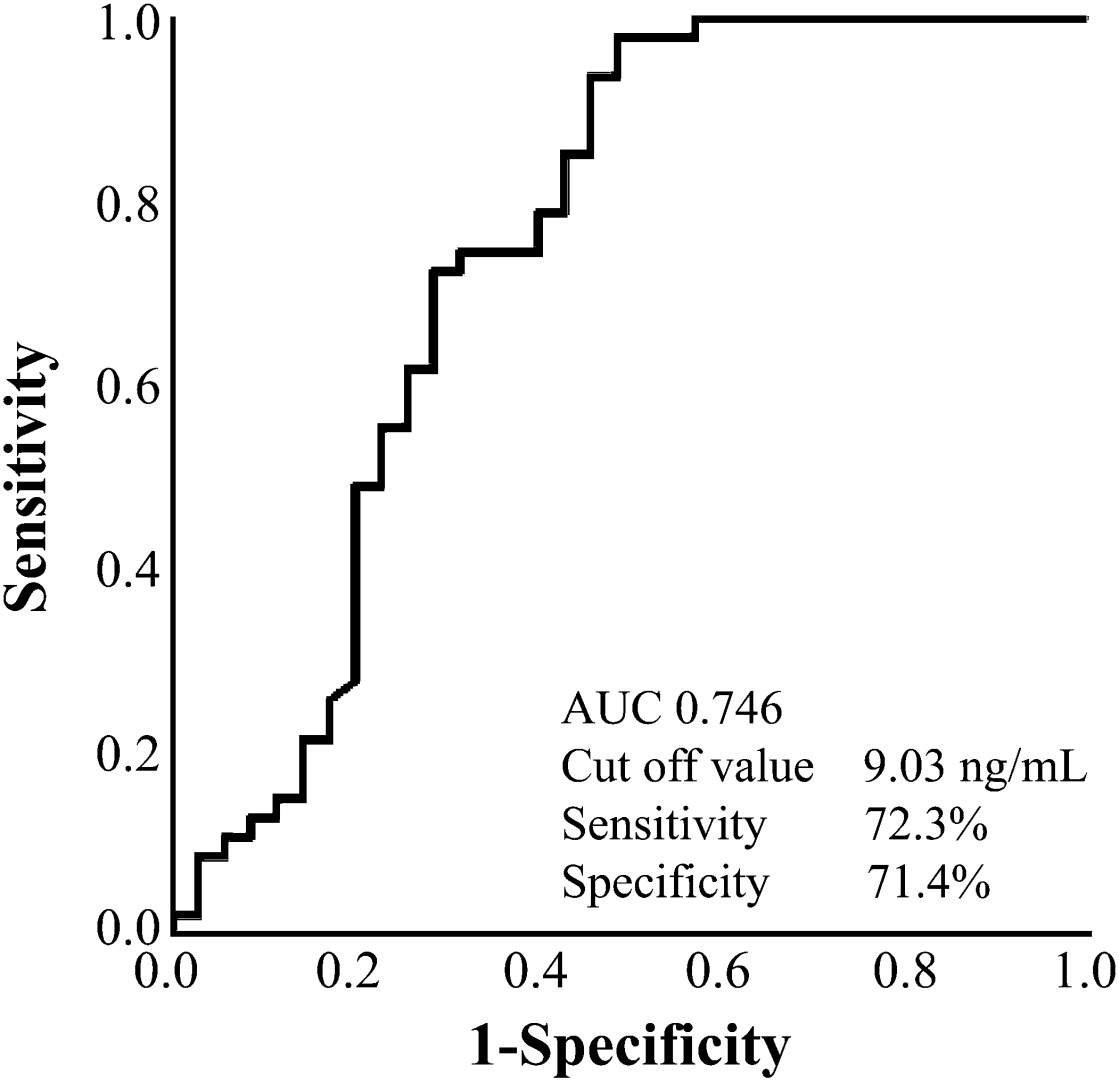
Receiver operating characteristic curve for SMRP for the differential diagnosis between BAPE and MPM. *BAPE* benign asbestos pleural effusion, *MPM* malignant pleural mesothelioma, *SMRP* soluble mesothelin-related peptides (SPSS Statistics V26 for IBM Japan (https://www.ibm.com/jp-ja/products/spss-statistics)).

When the ROC curve was drawn to confirm the reliability of SLPI to differentiate between BAPE and MPM and the cut-off value was 82.9 ng/mL, we found that the sensitivity was 82.4%, the specificity was 86.5%, and the AUC was 0.902, indicating that SLPI is an effective differential marker (Fig. 6). However, with a cut-off value of 47.1 μg/mL in the ROC curve for HA, the sensitivity was 77.0%, the specificity was 75.0%, and the AUC was 0.802 (Fig. 7), which, while useful, were inferior to those of SLPI. Furthermore, with a cut-off value of 9.0 ng/mL in the ROC curve for SMRP, the sensitivity was 72.3%, the specificity was 71.4%, and the AUC was below 8 (0.746; Fig. 8). In the ROC curve for CCL2, the cut-off was 1.8 pg/mL, the sensitivity was 62.7%, the specificity was 44.4%, and the AUC was 0.652. In the ROC curve for galectin-3, the cut-off was 11.4 ng/mL, the sensitivity was 67.9%, the specificity was 59.5%, and the AUC was 0.679. In the ROC curve for CYFRA21-1, the cut-off was 37.3, the sensitivity was 85.3%, and the specificity was 70.3. These results suggest that SLPI is a more effective marker than HA and SMRP, which are differential markers for mesothelioma, for the differential diagnosis of BAPE.

Tests of the ability of each pleural fluid marker to differentiate BAPE from early-stage MPM revealed that, SLPI, CYFRA21-1, HA and SMRP can be used in the diagnosis of BAPE. A combination of SLPI and CYFRA21-1 for the differentiation between BAPE and early-stage MPM achieved an AUC of 0.965.

## Discussion

By focusing on BAPE and differentiating it from early-stage MPM and other diseases, we found that SLPI in pleural fluid was a significant indicator because the values were significantly lower than those for other diseases. We believe that the mechanism of BAPE (i.e., asbestos fibres on the visceral pleura) may involve mechanical inflammation without wound healing and the prevention of tissue destruction because of the low SLPI value observed in pleural effusion.

Although HA and SMRP reportedly serve as differential markers for MPM and other diseases, upon drawing the ROC curve, SLPI had an AUC of 0.902, indicating higher reliability than CYFRA21-1, HA and SMRP.

Combining pleural effusion markers with pleural fluid cytology, chest CT, and positron emission tomography–CT images facilitates the differentiation of BAPE from MPM and LCa, which are malignant tumours. However, in the early stages of MPM, many patients do not exhibit significant uptake on chest CT or PET-CT, and it has been reported that MPM is diagnosed by searching for the presence of p16 gene mutations in histopathology specimens using FISH and cytology tools when more than a certain number of homozygous deletions are confirmed^2,12^. However, in MPM patients, the rate of diagnosis by pleural effusion cytology is much lower than that by pleural effusion caused by malignant tumours such as LCa. Therefore, even if tumour cells are detected, markers that suggest MPM is present. In the past, such markers included osteopontin^13^ and fibulin-3^14,15^, but at present, they are rarely evaluated.

As a marker for differentiating pleural effusion in MPM, SLPI is described only in a report by Blanquart et al.^16^; however, in their report, three markers (CCL2, galectin-3, and SMRP) were reportedly effective, and if they were used properly, MPM could be differentiated from other diseases that cause pleural effusion, with an AUC of 0.968. However, Blanquart et al. used three markers rather than a single marker. For SLPI alone, the AUC was 0.706, which was the lowest among the markers examined, but its significance was not evaluated. However, our data showed that for the differentiation between BAPE and early-stage MPM, the AUC of a single marker (SLPI) was 0.902, and its combination with CYFRA21-1 achieved an AUC of 0.965, close to the 3 markers described above.

With regard to MPM, CCL2 levels in pleural fluid are high and reportedly increase as the disease progresses^17^. We reported that high levels were found in the serum in patients with advanced-stage MPM^18^. In the present study, we examined CCL2 in pleural fluid and found significantly higher levels in patients with BAPE than in patients with LCa and other diseases. However, with respect to MPM, which should be associated with high levels, we found significantly lower levels compared with BAPE (p < 0.016), in contrast to Blanquart et al.^16^, who reported that the levels differed according to the histological type, with 2.82 ng/mL in epithelial mesothelioma and 16.73 ng/mL in sarcomatoid mesothelioma. Our patients included 31 with epithelial mesothelioma and six with sarcomatoid mesothelioma (indicating overwhelmingly more patients with epithelial mesothelioma), and the mean level was therefore low, at 2.15 pg/mL. Of the 37 MPM patients included in this study, three had sarcomatoid mesothelioma, and while some patients had a high level, the level in patients with epithelial mesothelioma was 0.4 to 3.0 pg/mL, indicating significant individual variation. We therefore intend to conduct another study with a larger sample size.

Galectin-3 levels were not only high in MPM patients but also in LCa patients, and we therefore suspect that galectin-3 can be used to rule out malignancy because low levels are found in BAPE patients^19^. Similarly, for CYFRA21-1, high levels are common in pleural effusions, even in early-stage MPM, and therefore, even if there is no malignant pleural thickening on imaging, early-stage MPM should be considered because CYFRA21-1 appears to be a marker that can warrant thoracoscopic biopsy^20^.

When comparing HA, which is a biomarker of mesothelioma, and SMRP by focusing not on MPM but on BAPE, we found a significantly high AUC of 0.902 for SLPI and significantly lower AUCs for CYFRA21-1 (0.853), HA (0.802) and SMRP (0.746). Even on its own, SLPI was deemed a superior marker for differentiating BAPE from MPM compared with HA and SMRP. We also found that it was more useful than HF or IF for differentiating LCa. A differential diagnosis of LCa can be based on cytology or tumour markers such as carcinoembryonic antigen and CYFRA21-1; for tuberculosis pleurisy among IF, it can be based on adenosine deaminase; and for inflammatory pleurisy, a differential diagnosis can be achieved based on neutrophilia in pleural fluid.

Because SLPI levels were significantly lower in patients with BAPE than in patients with pleurisy caused by other diseases, such as MPM, SLPI may be an effective screening marker for the diagnosis of BAPE and in the differential diagnosis of early-stage MPM.

## Conclusions

The levels of SLPI in the pleural fluid of patients with BAPE were significantly lower than those in patients with MPM, LCa, and other diseases. For the differential diagnosis of early-stage MPM, we propose the inclusion of SLPI as a pleural effusion marker along with CYFRA21-1.

## Data Availability

All data generated or analysed during this study are included in this published article.

## Abbreviations

AUC: Area under the curve
BAPE: Benign asbestos pleural effusion
BRCA1: Breast cancer susceptibility gene I
CCL2: C–C motif chemokine ligand 2
CYFRA21-1: Cytokeratin 19 fragment 21-1
CT: Computed tomography
FISH: Fluorescence in situ hybridization
HA: Hyaluronic acid
HF: Heart failure
IF: Infection
LCa: Lung cancer
MCP1: Monocyte chemotactic factor 1
MPM: Malignant pleural mesothelioma
ROC: Receiver operating characteristic
SLPI: Secretory leukocyte peptidase inhibitor
SMRP: Soluble mesothelin-related peptides
